# Supplementary figures and images for: Symbiont-induced odorant binding proteins mediate insect host hematopoiesis
Source: eLife. 2017 Jan 12;6:e19535. doi: 10.7554/eLife.19535 (PMC5231409; doi:10.7554/eLife.19535)

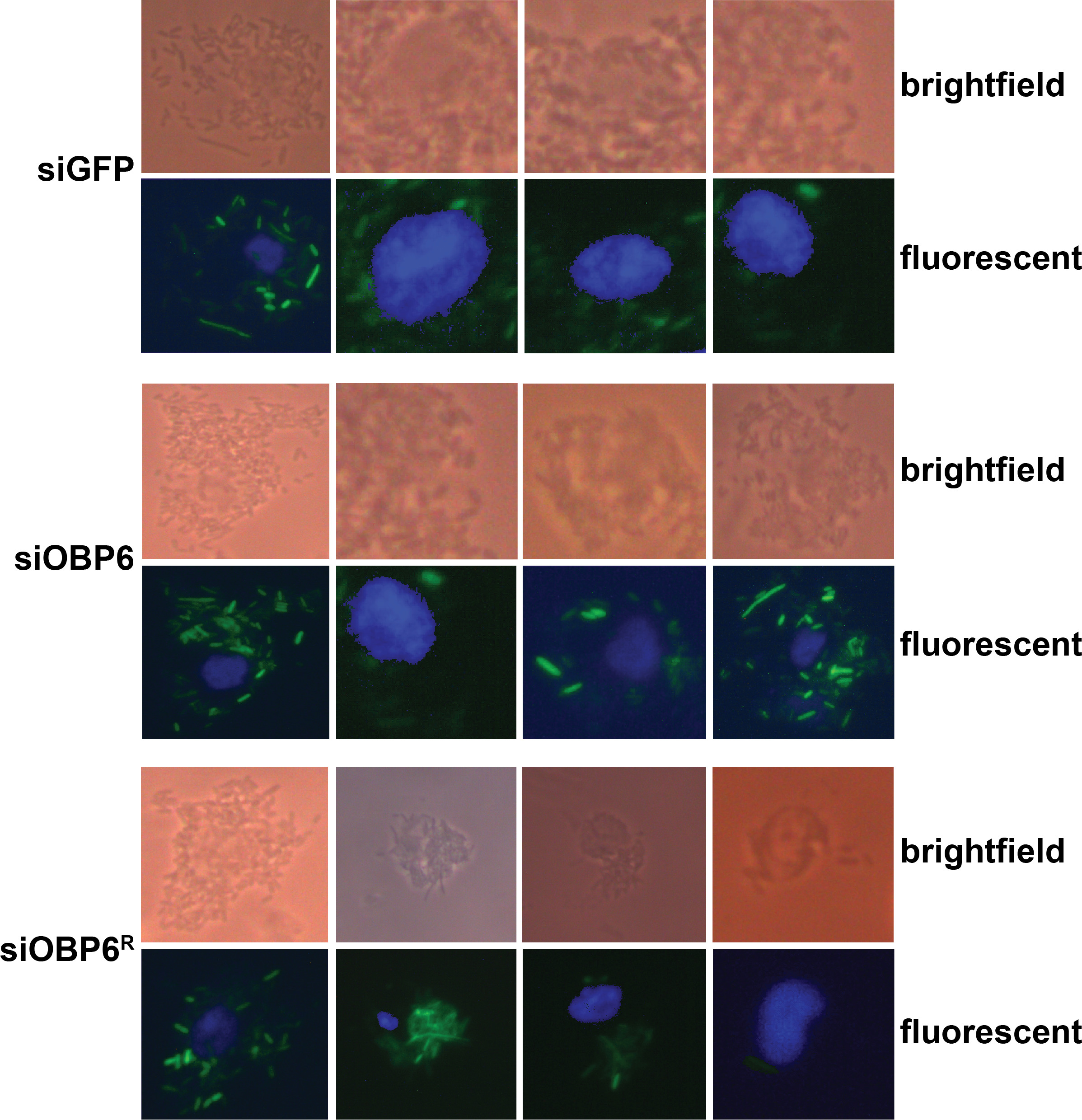

Supplement: Figure 2—source data 2. — DOI: http://dx.doi.org/10.7554/eLife.19535.008 [file elife-19535-fig2-data2.jpg]

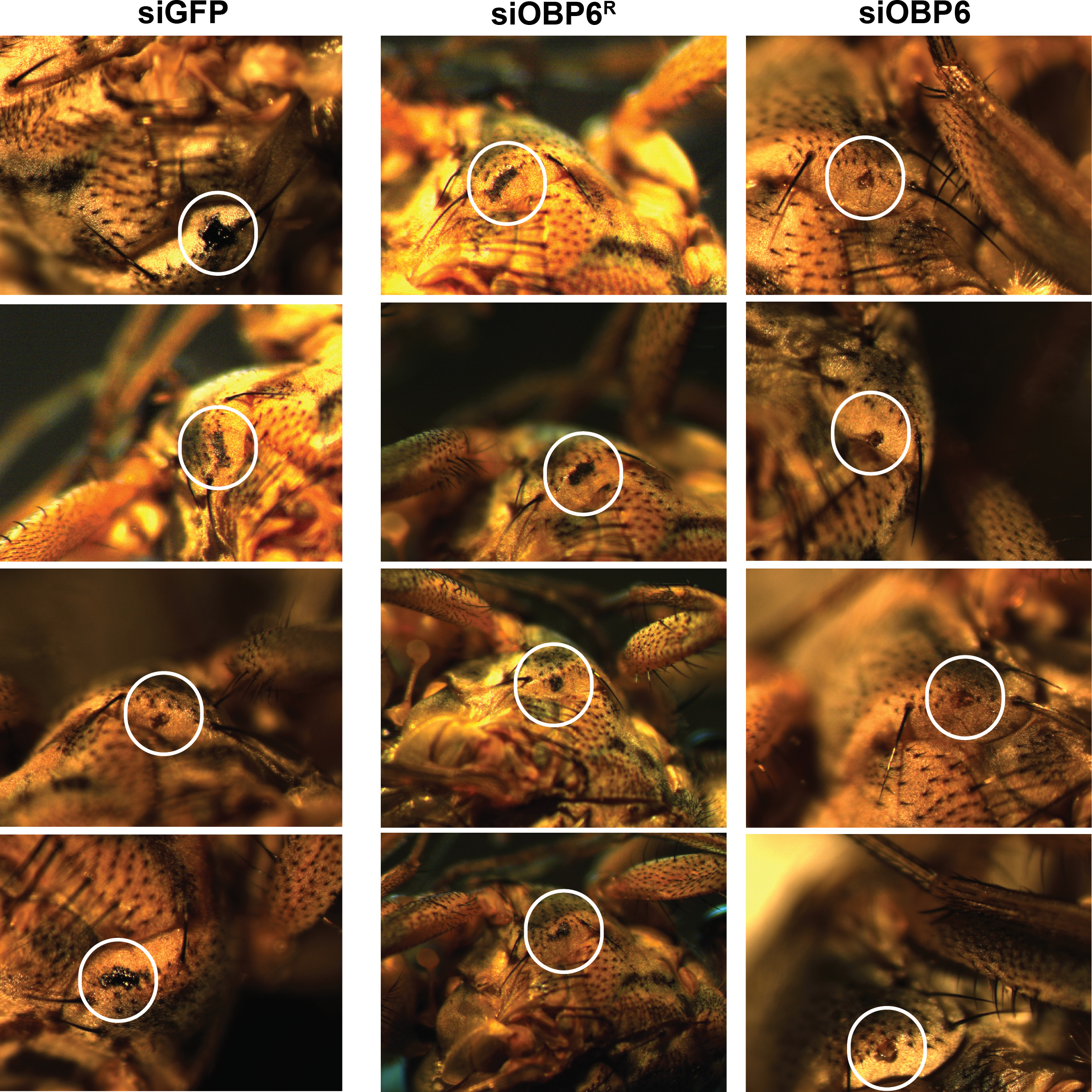

Supplement: Figure 3—source data 1. — DOI: http://dx.doi.org/10.7554/eLife.19535.011 [file elife-19535-fig3-data1.jpg]

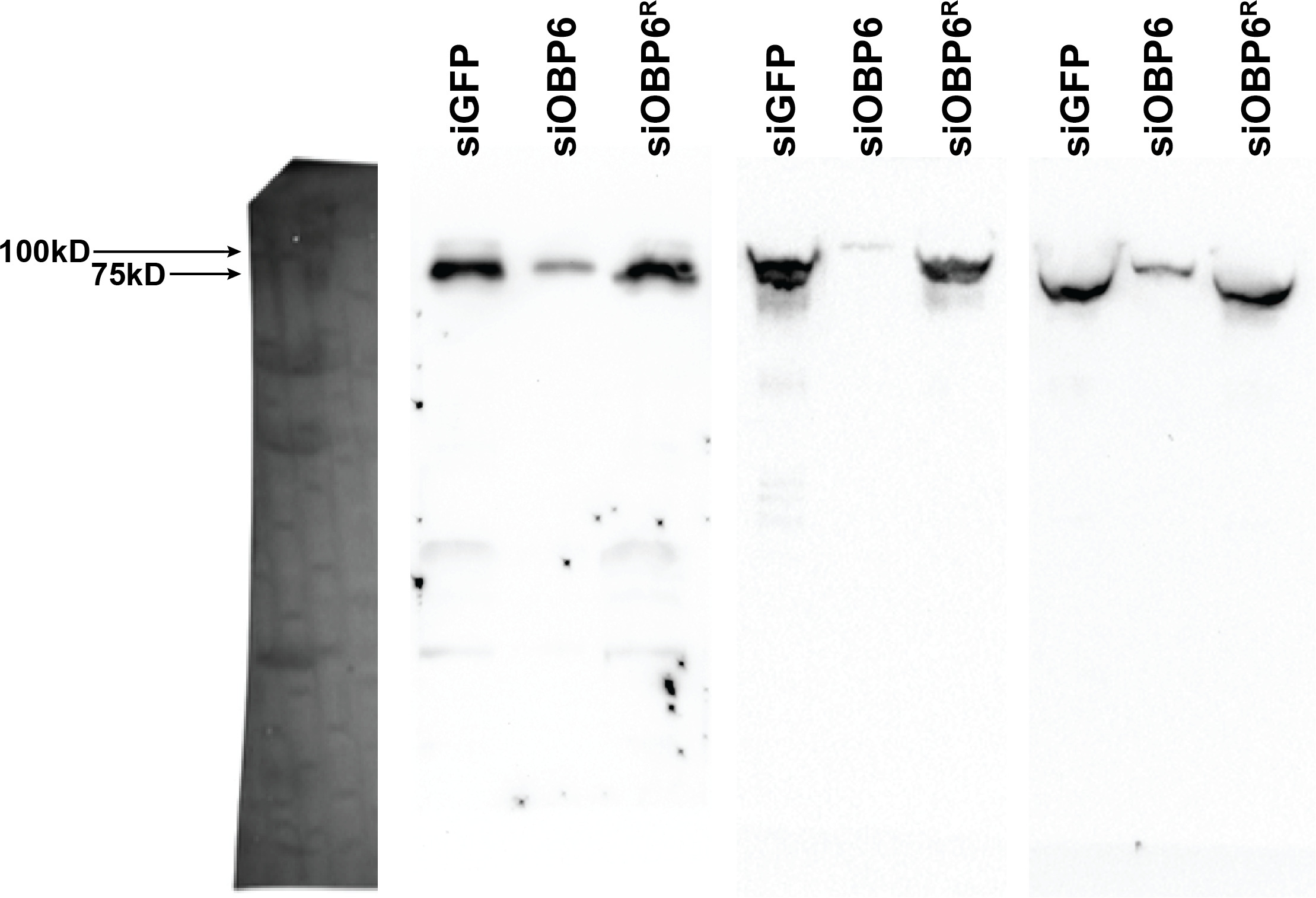

Supplement: Figure 3—source data 2. — DOI: http://dx.doi.org/10.7554/eLife.19535.012 [file elife-19535-fig3-data2.jpg]

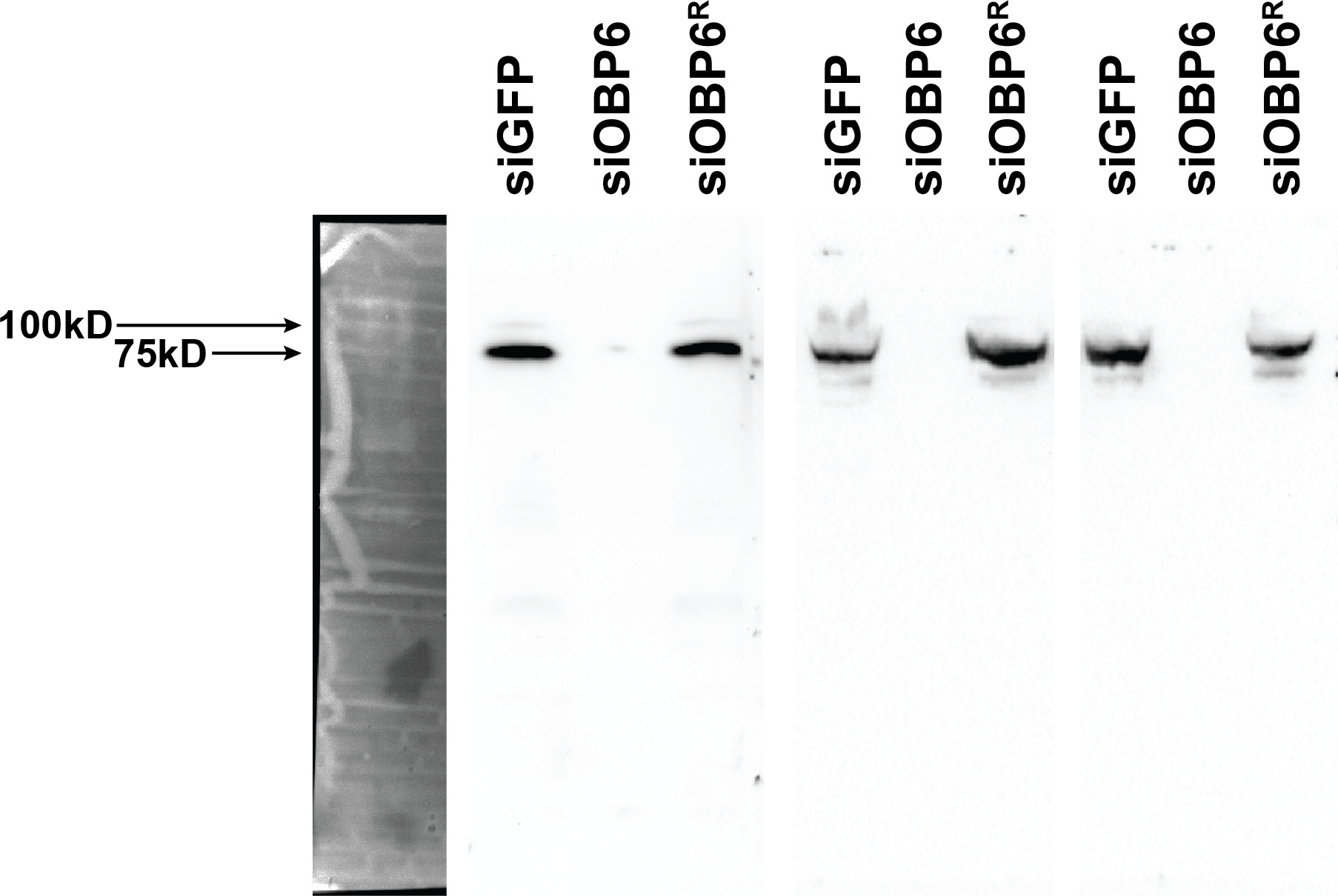

Supplement: Figure 3—source data 3. — DOI: http://dx.doi.org/10.7554/eLife.19535.013 [file elife-19535-fig3-data3.jpg]

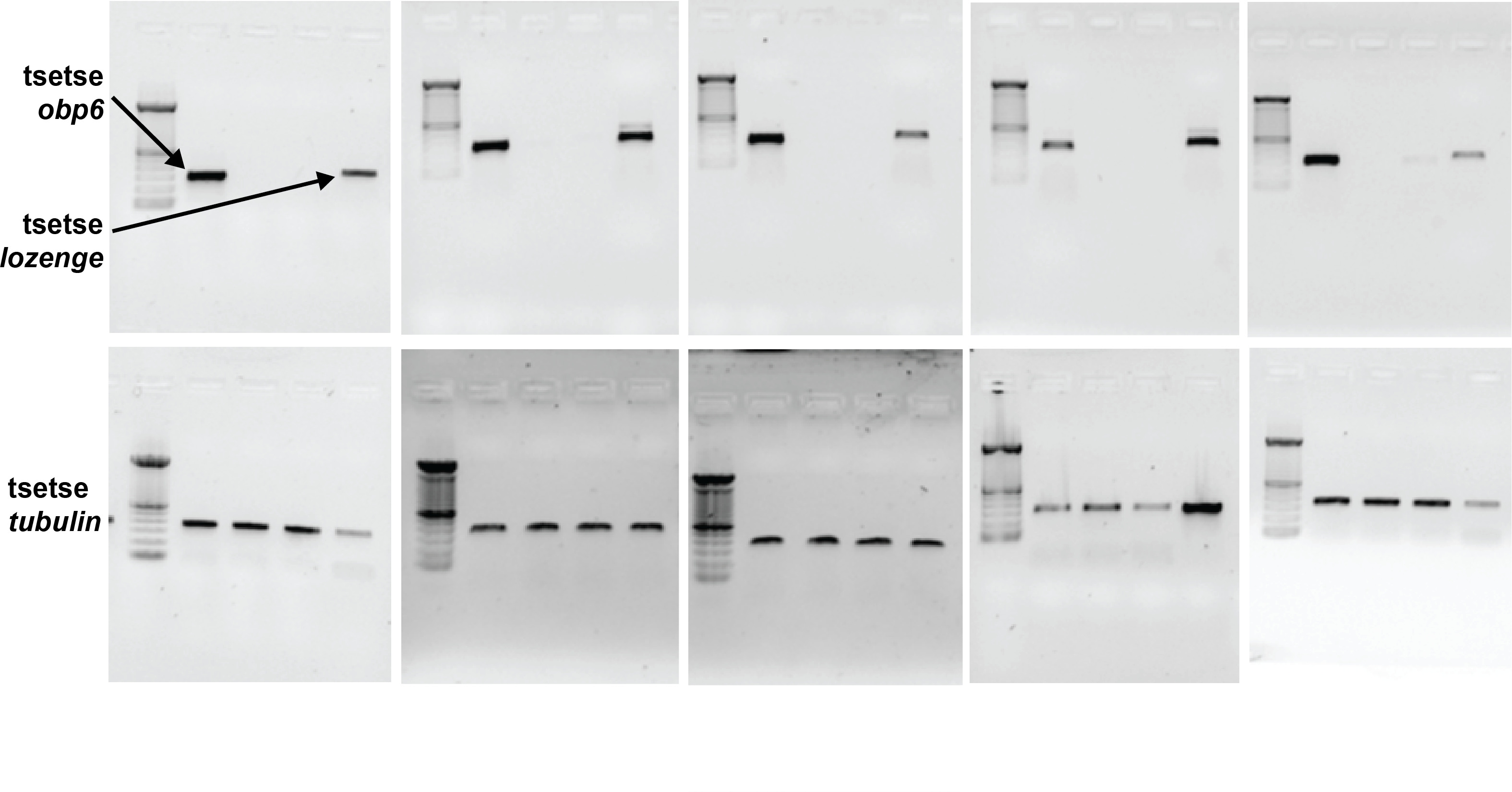

Supplement: Figure 4—source data 3. — DOI: http://dx.doi.org/10.7554/eLife.19535.018 [file elife-19535-fig4-data3.jpg]

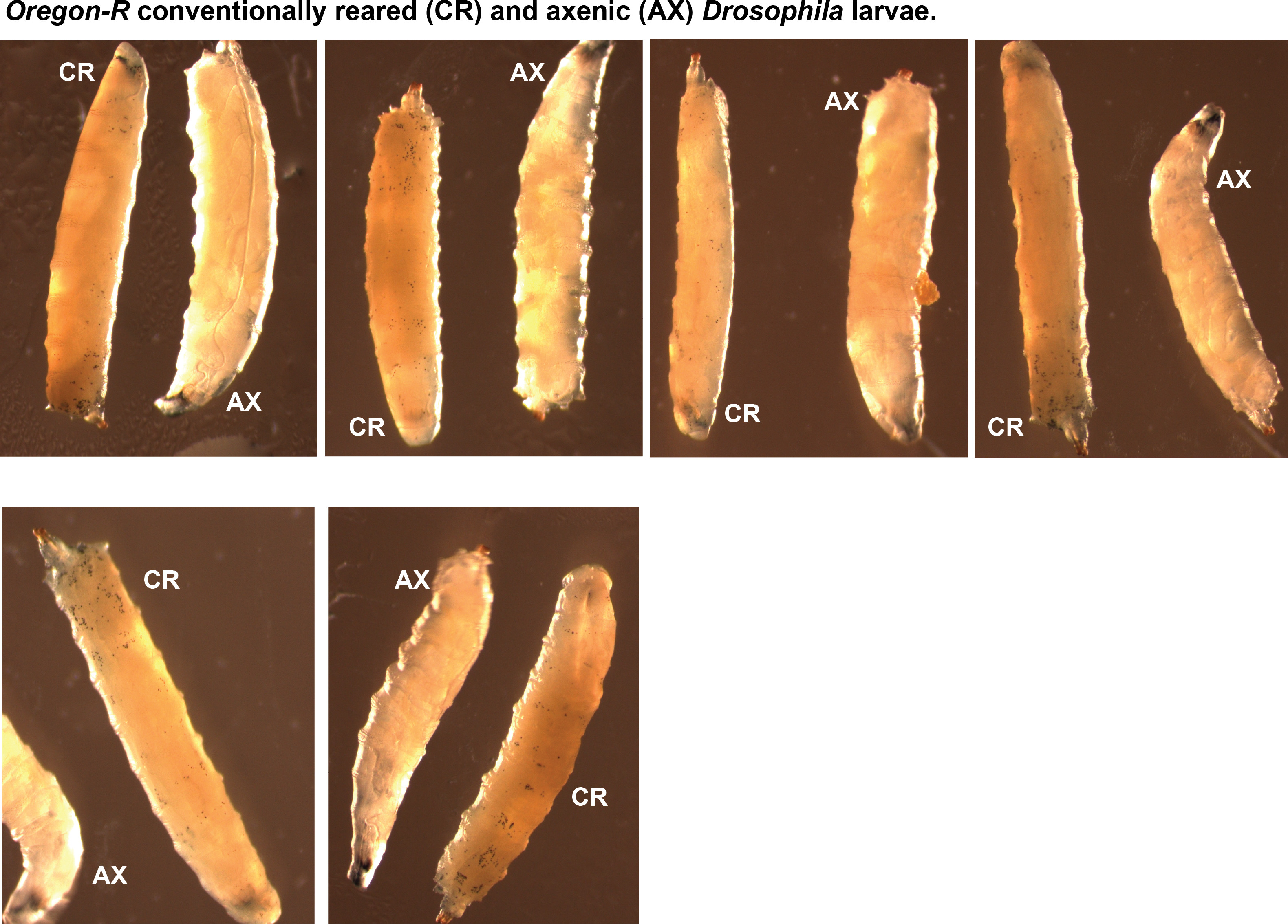

Supplement: Figure 5—source data 2. — DOI: http://dx.doi.org/10.7554/eLife.19535.021 [file elife-19535-fig5-data2.jpg]

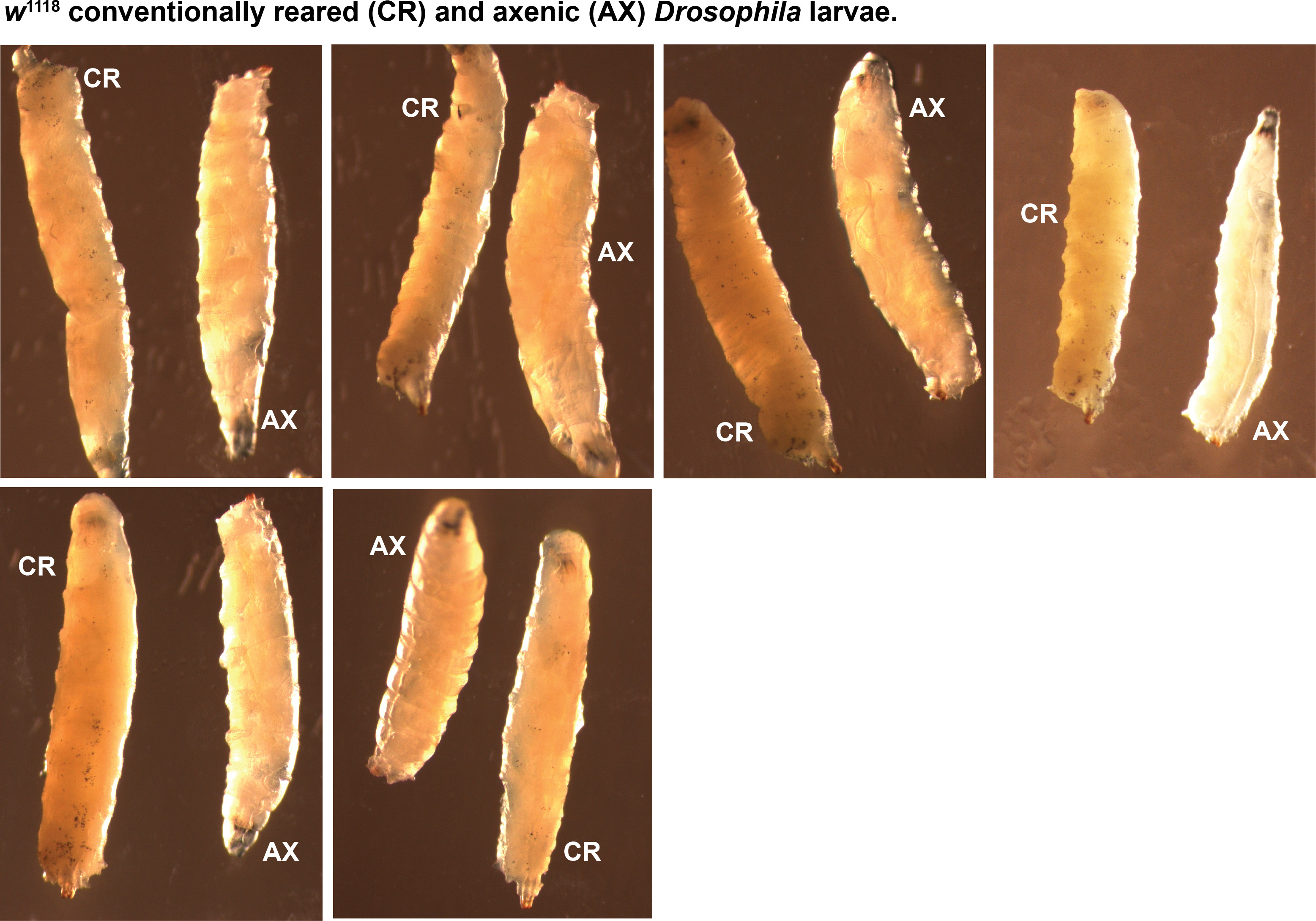

Supplement: Figure 5—source data 3. — DOI: http://dx.doi.org/10.7554/eLife.19535.022 [file elife-19535-fig5-data3.jpg]

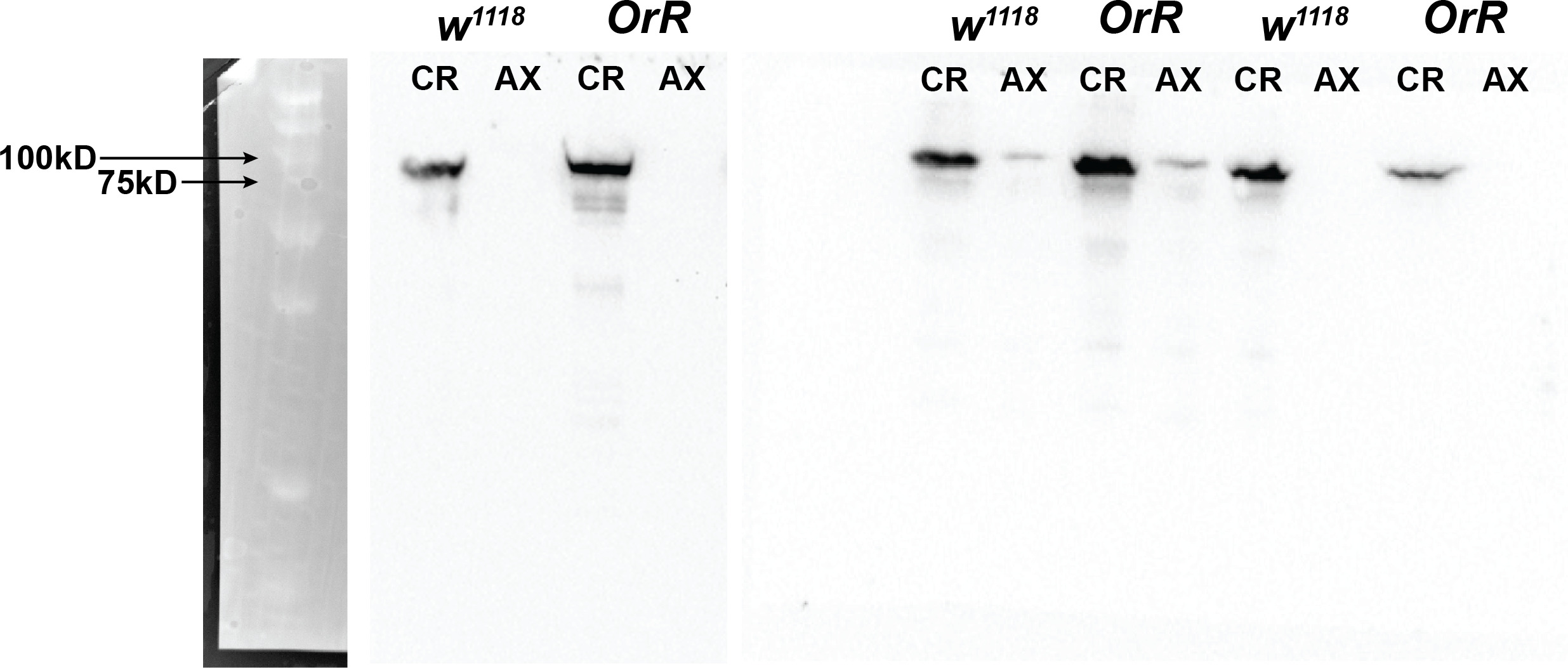

Supplement: Figure 5—source data 4. — DOI: http://dx.doi.org/10.7554/eLife.19535.023 [file elife-19535-fig5-data4.jpg]

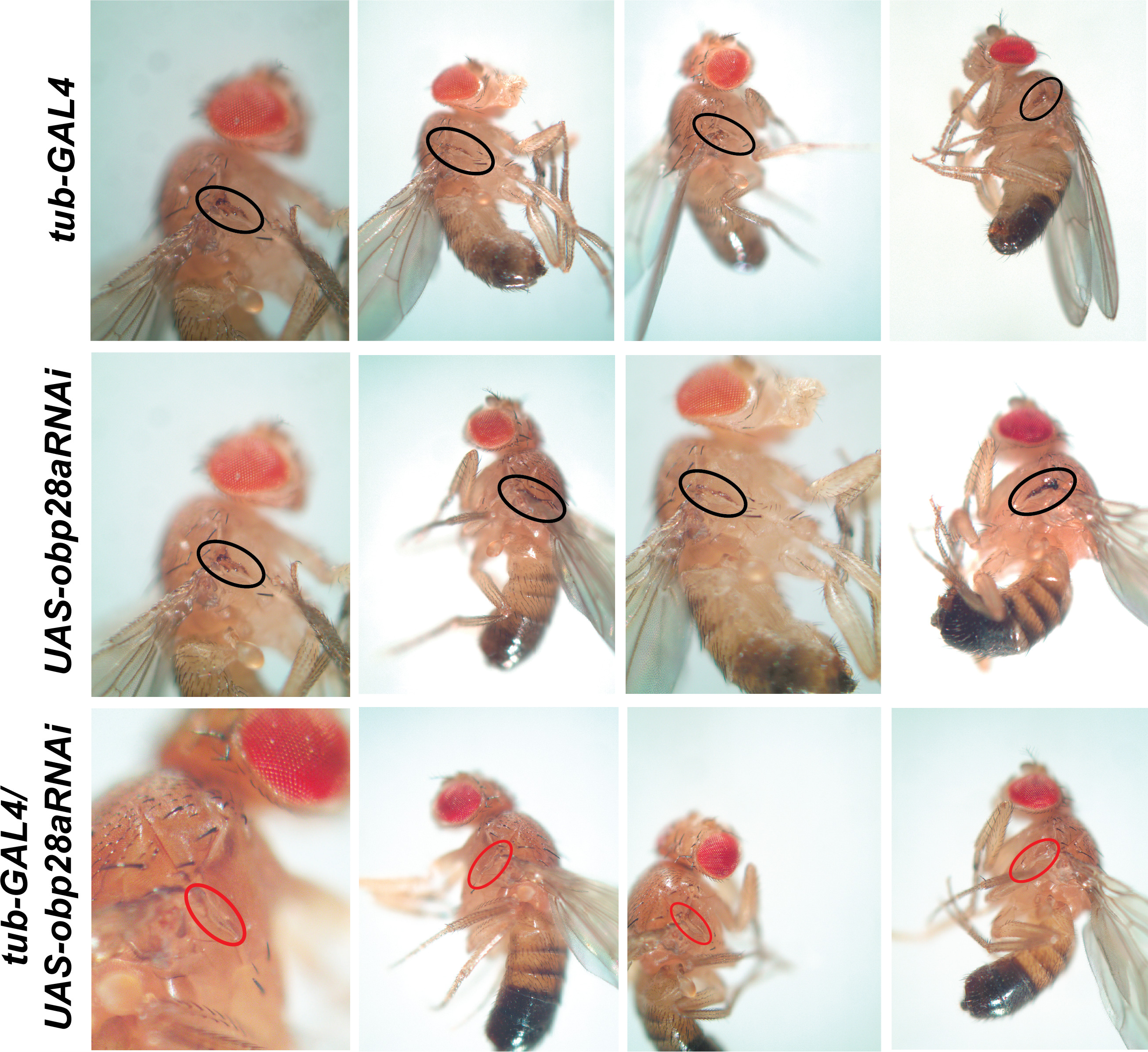

Supplement: Figure 5—source data 5. — DOI: http://dx.doi.org/10.7554/eLife.19535.024 [file elife-19535-fig5-data5.jpg]

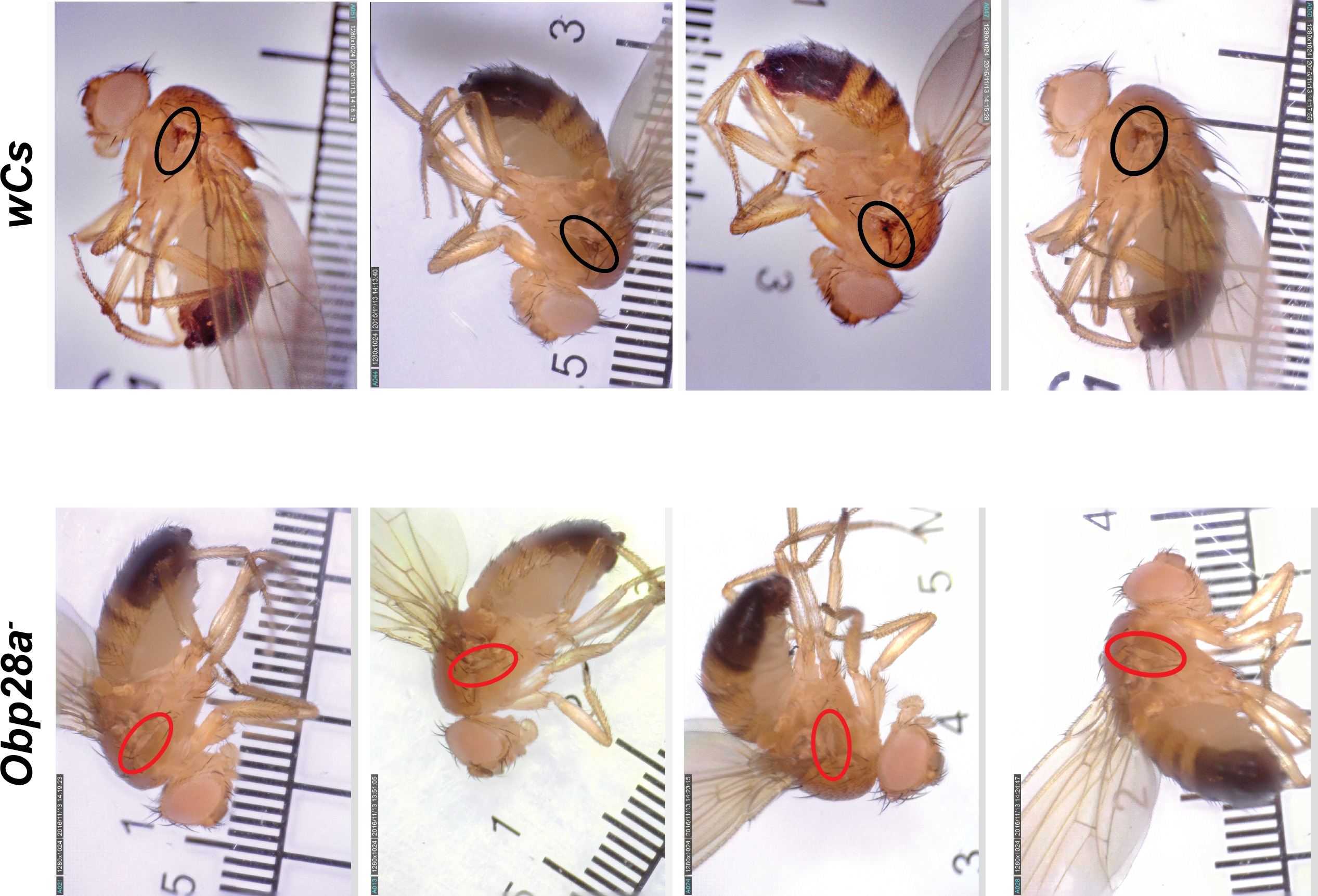

Supplement: Figure 5—source data 6. — DOI: http://dx.doi.org/10.7554/eLife.19535.025 [file elife-19535-fig5-data6.jpg]

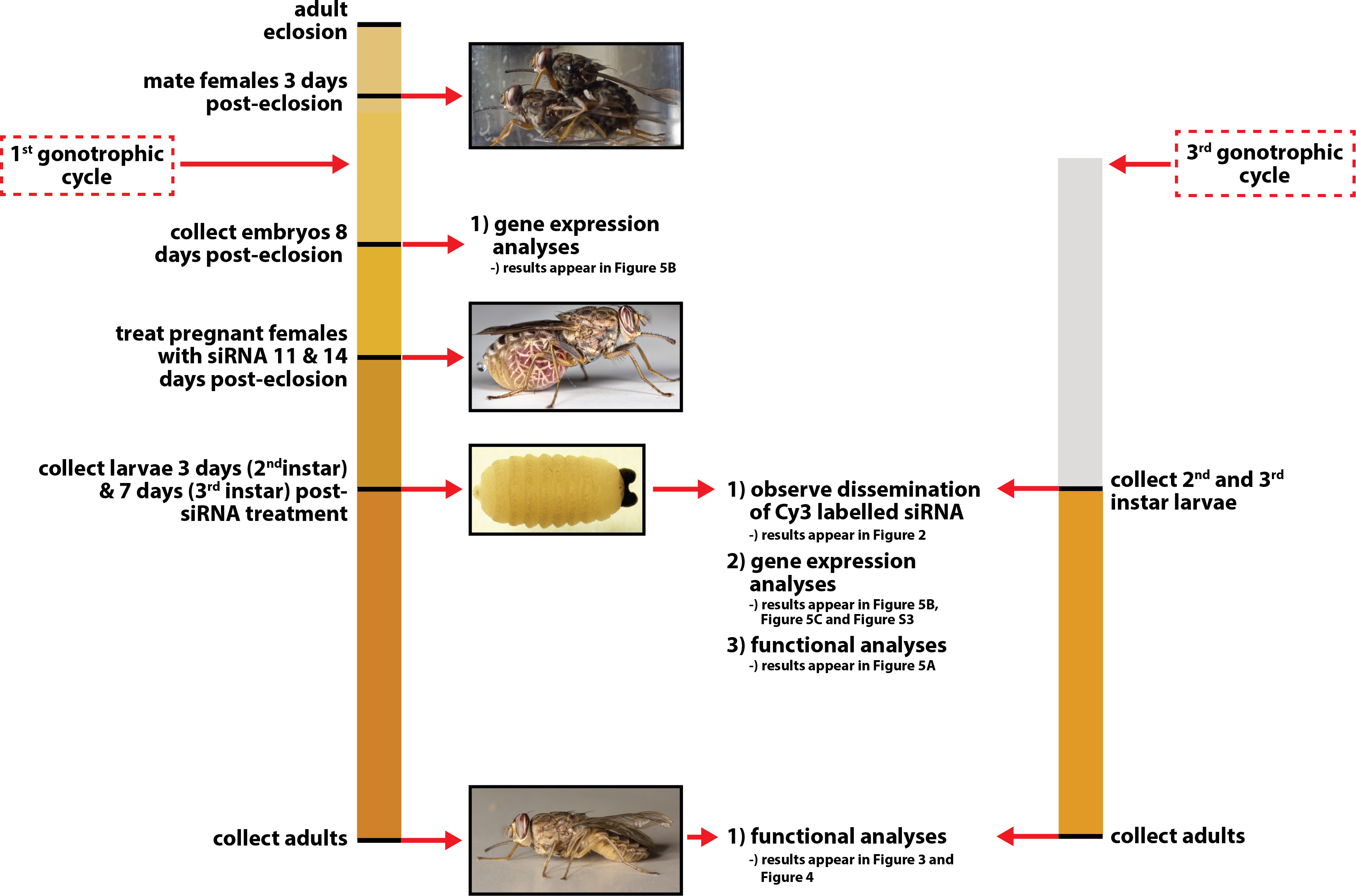

Supplement: Supplementary file 3. — All relevant experimental details are described in the Materials and methods, under the ‘RNA interference’ sub-heading. DOI: http://dx.doi.org/10.7554/eLife.19535.030 [file elife-19535-supp3.jpg]

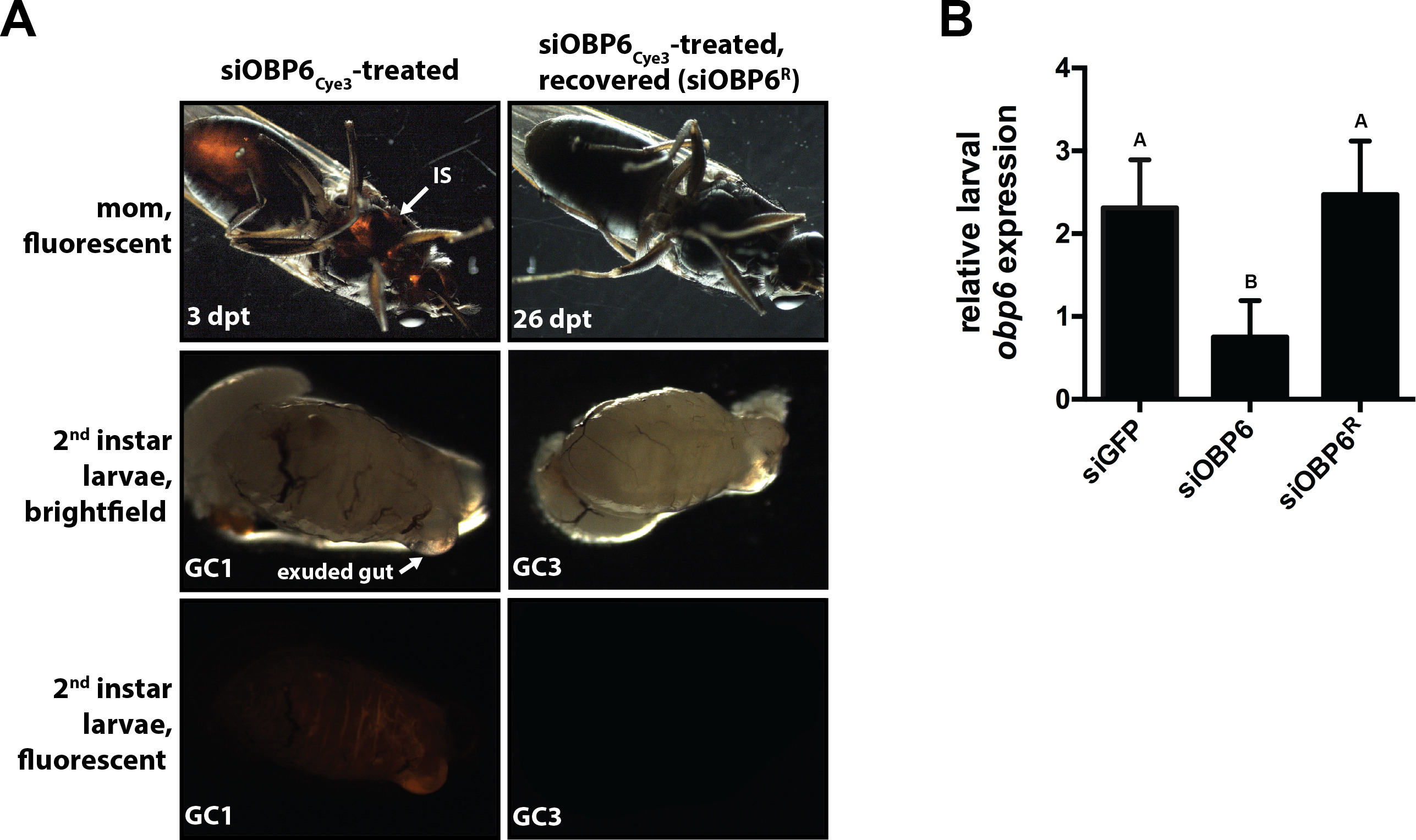

Supplement: Supplementary file 4. — (A) Pregnant females were intra-thoracically injected with Cy3 tagged anti-obp6 short interfering (si) RNAs (siOBP6Cy3). A representative micrograph showing that siOBP6Cy3 had disseminated throughout the maternal hemocoel by three days post-treatment (dpt; top left panel), and was present in second instar larvae from the first gonotrophic cycle (GC1; middle left panel) of these females as visualized using fluorescent illumination (bottom left panel). By 26 dpt, siOBP6Cy3 was absent from treated moms (top right panel) and their third gonotrophic cycle (GC3) larvae (middle and bottom right panels). Five pregnant treatment and recovered females were visualized to observe siOBP6Cy3 dissemination and transfer to larvae. (B) Effectiveness of siRNA-based obp6 knockdown in intrauterine second instar tsetse larvae. Relative expression of obp6 in second instar siGFP, siOBP6 and siOBP6R intrauterine larvae. RT-qPCR analysis was performed using larvae from two distinct experiments, each of which included 4 (siGFP and siOBP6) or 3 (siOBP6) biological replicates (each consisting of a mixture of four first and second instar larvae). All RT-qPCR results were normalized relative to tsetse's constitutively expressed β-tubulin gene (determined from each corresponding sample). Data are presented as mean of all replicates from both experiments, ± SEM. Bars with different letters indicate a statistically significant difference (p<0.05) between treatments. Statistical analysis = 2 way ANOVA. DOI: http://dx.doi.org/10.7554/eLife.19535.031 [file elife-19535-supp4.jpg]
